# Long-term impact of self-financed rotavirus vaccines on rotavirus-associated hospitalizations and costs in the Valencia Region, Spain

**DOI:** 10.1186/s12879-017-2380-2

**Published:** 2017-04-11

**Authors:** Alejandro Orrico-Sanchez, Mónica López-Lacort, Silvia Pérez-Vilar, Javier Díez-Domingo

**Affiliations:** 1 Vaccine Research, Fundación para el Fomento de la Investigación Sanitaria y Biomédica de la Comunidad Valenciana, FISABIO-Public Health, Avenida de Cataluña 21, CP, 46020 Valencia, Spain; 2 Universidad Católica de Valencia ‘San Vicente Martir’, Valencia, Spain

**Keywords:** Rotavirus vaccine, Spain, Hospitalization, Impact, Costs, Statistics, Child

## Abstract

**Background:**

Rotavirus vaccines are available in Spain from 2007. They are recommended by the Spanish Pediatric Association, but not funded by the National Health System (NHS) and its coverage rate reached 40-50%. The hospitalization rate reduction of rotavirus caused gastroenteritis (RVAGE) directly attributable to vaccination remains unclear due to the large differences described in published studies, ranging from 14 to 44.5% in children <5 years of age, even with similar vaccination coverage. These results could be partly explained by variability in hospitalization policies, different study designs and the timeframe of observation. In addition, the direct economic impact of the reduction of hospitalizations has never been estimated. Therefore, there is a need to analyze the long-term impact of rotavirus vaccines on RVAGE and all cause gastroenteritis (AGE) hospitalizations and the national health system associated costs, minimizing potential confounders or biases.

**Methods:**

A population-based, ecological study using the hospital discharge registry’s Minimum Basic Data Set (MBDS) and the vaccine register (SIV) was performed, among Valencia Region’s children <5 years old, during 2002 - 2015. RVAGE and AGE hospitalization risk was analyzed by vaccine coverage and adjusted by the total hospitalization rate for all causes to avoid external biases. The impact of AGE-associated health care utilization in prevaccine (2003–2006) versus postvaccine (2008–2014) years was also assessed.

**Results:**

After vaccines licensure, the incidence of RVAGE-associated hospitalizations decreased markedly. A general vaccine coverage-related reduction in RVAGE or AGE-hospitalizations risk was observed in all age groups. Compared with unvaccinated children, RVAGE hospitalization risk decreased by 67% (95% CI: 55-67), 71% (95% CI: 58-81) and 68% (95% CI: 18-92) in children 0, 1 and 4 years of age, respectively, with a vaccination coverage between 40 and 42%. Overall, the hospital related costs were reduced around EUR 6 Mill per 10^5^ children in 7 years.

**Conclusions:**

Despite the low-medium vaccine coverage, the introduction of rotavirus vaccines had a specific coverage-related response impact in the hospitalizations for RVAGE and AGE in children <5 years and their use substantially reduced hospital related costs. The model used reassures that the estimated impact is due to the vaccination and not to other external factors.

**Electronic supplementary material:**

The online version of this article (doi:10.1186/s12879-017-2380-2) contains supplementary material, which is available to authorized users.

## Background

Rotavirus (RV) is the most frequent cause of acute gastroenteritis (AGE) in young children worldwide. It is estimated that by the age of five, nearly every child has been infected at least once by a rotavirus [1]. An estimated 75,000 children aged <5 years are hospitalized for rotavirus acute gastroenteritis (RVAGE) each year in European countries, leading to high demands on health care systems [2].

Two rotavirus vaccines, the monovalent (RV1; Rotarix®, GSK) and the pentavalent (RV5; RotaTeq®, Merck), were licensed in Spain in August 2006 and January 2007, respectively. Although institutions such as The World Health Organization (WHO) and The Advisory Committee on Immunization Practices (ACIP) recommend the inclusion of rotavirus vaccination in national immunization programmes [1, 3], they are not funded by the Spanish National Health System (NHS). However, they are recommended by the Spanish Paediatric Association, and paid by parents reaching a coverage rate of around 40-50% [4–6]. Due to the incidental finding of circovirus DNA contamination in both vaccines, the Spanish Medicines Agency suspended RV5 (from June 2010 to November 2010) and RV1 (February 2010 to June 2016) distribution.

The Valencia Region of Spain has a population of 5 million and represents 10% of the Spanish population. Studies in this region showed that rotavirus was responsible for 53% of all gastroenteritis hospitalizations in children aged <5 years before vaccination [7], and had a vaccine effectiveness around 80% [8], but the evaluation of the vaccine impact, by examining incidence and trends in RVAGE and all-cause AGE-hospitalizations before and after vaccination has not yet been examined, and organizations like WHO considers it necessary [1].

Impact of RV vaccines in hospitalizations has already been described in different countries [4, 9–11]. However, 9 years after the introduction of rotavirus vaccines in Spain, the proportion of the RVAGE-hospitalization rate reduction directly attributable to vaccination remains unclear due to the large variation in the rate reductions found between studies, ranging from 14 to 44.5% and 10 to 57% in children <5 and <1 years of age, respectively, even with similar vaccination coverage [4–6, 12]. Along the same lines, wide differences in the annual incidence of RVAGE and all-cause AGE-hospitalizations were also found [4–6, 12]. This variability supposes a limitation to make comparisons and creates uncertainty when making decisions about the potential inclusion of the vaccine in the official immunization schedule. This disparity in results could be partly explained by the different geographic areas among studies, but other important differences in the study design could also have influenced. First, variability in hospitalization policies or other external variations such as a global financial and economic crisis that clearly affected health systems [13]. Second, methodological differences and/or deficiencies in the analytical method used and the treatment of the potential confounders could also bias the results. Finally, the observation period, that may alter the results if too short, and, as other authors pointed out, studies with extended study periods should be done [6].

Therefore, there is a need to carry out long-term impact studies of vaccination, designed in such a way that the results could be directly attributed to the vaccine by minimizing potential confounders or biases.

In high income countries RV has a major family and social impact [14] with minimal mortality [15]. It was estimated that the annual cost for the Spanish national health system is approximately €28 million, and causes productivity loss in two thirds of parents [16]. However, limited information is available regarding the direct economic impact of the vaccination.

The main objective of the study is to assess the long-term impact of rotavirus vaccines on RVAGE-hospitalizations among children under 5 years of age residing in the Region of Valencia. Secondary aim is to evaluate the economic impact of rotavirus vaccines on all-cause AGE-hospitalizations and the national health system associated costs.

These results could better inform decisions about its potential inclusion in the official immunization schedule.

## Methods

### Study settings and population

Using routinely collected health data, we performed a population-based ecological study in the Valencia Region. The study included all children residents in the Region born from January 1st 2002 through December 31st 2014, aged less than 5 years, and the study period from 1st January 2002 until 30th September 2015.

The Valencia Region has a population of approximately 5,000,000 inhabitants, and an annual birth cohort of 48,000 infants. Approximately 98.3% of the population is covered by the public health system [17]. The regional health system is currently divided into 24 Departments, which includes 24 paediatric hospitals. However, the distribution of departments has changed over the study period. Thus, we have considered for analysis the structure of 2002 consisting of 20 departments, finally reduced to 19 because one had no paediatric section at the hospital and children were derived to the closest department.

### Data sources

The population data was obtained from the regional population-based administrative database (SIP) that collects and updates demographic data, health services assignment and use of the NHS [18]. For hospitalization we used the hospital discharge database, MBDS (minimum basic data set) [8], where discharged diagnoses are ICD-9-CM coded. Vaccination coverage was obtained from the regional vaccine information system (SIV), where administered doses are registered [8].

#### Case definition

Acute gastroenteritis-associated hospitalizations were codified in MBDS with the ICD-9-CM codes: 001-009 (intestinal infectious diseases), 558.9 (other and unspecified non-infectious gastroenteritis and colitis) and 787.91 (diarrhoea not otherwise specified).

Our outcomes were: (a) rotavirus acute gastroenteritis (RVAGE) hospitalization, defined as hospitalization with a discharge diagnosis of enteritis due to rotavirus (ICD-9-CM code 008.61) in any diagnosis position and (b) acute gastroenteritis (AGE) hospitalization, defined as hospitalization with a discharge diagnosis of gastroenteritis-associated episode (ICD-9-CM codes 001-009, 558.9, 787.91) in any diagnosis position.

### Vaccine coverage

Vaccine coverage was defined as the proportion of the study population vaccinated with at least one dose of RV1 or RV5, with no distinction between vaccines.

### Statistical analysis

Database administrators provided the population, vaccination and hospitalizations aggregated by gender, age, year, month and health department.

RVAGE and AGE hospitalization rates and the impact of vaccination were analyzed for five age groups (0,1,2,3 and 4 years) by a Bayesian mixed Poisson regression, adjusted by gender, vaccine coverage (stratified in 4 groups: 0%, 1-19%, 20-39% and ≥40%), health department (as a random effect), the time as continuous variable that indicated time in months at time t from the start of the observation period, other time variable that counted the months after intervention (code 0 for the pre-vaccination period), the month of the year to control seasonality and the id. variable (corresponding with records from the database) as a random effect to avoid over-dispersion [15]. In addition, the model was adjusted by the total hospitalization rate for all causes except AGE (in quintiles) to avoid external biases.

The impact of vaccination was calculated as the risk ratios (RR) of hospitalizations for RVAGE or AGE by age group and by vaccination coverage. Results are presented with 95% credible intervals (CrI) that are analogue to 95% confidence intervals in frequentist statistics.

The AGE costs for the health-care system were calculated by the total days of hospital stay per year divided by the population of each year. The cost reduction between pre- and post-vaccination periods (2003-2006 vs 2007, 2008,.., 2014) was estimated by a negative binomial regression adjusted by the total days of hospital stay rate except AGE, gender, health department, age and month. We considered the unit cost for 1 day of hospital care in a paediatric ward (€544.08) in The Valencia Region [19].

Analyses were carried out using R Statistical Software (Foundation for Statistical Computing, Vienna, Austria) and WinBUGS.

## Results

### Characteristics of the study population

Of the 322,255 hospitalizations in children <5 years of age, 24,021 (˷7,5%) had an AGE diagnosis code. Among them, 6076 were codified as RVAGE, representing 25.3% of AGE hospitalizations (Table 1).

**Table 1:** Descriptive analysis of cumulative hospitalizations for RVAGE, AGE and total hospitalizations in children <5 years old of The Region of Valencia between 2002 and 2015

	RVAGE-hospitalizations			AGE-hospitalizations			All-cause hospitalizations		
Age (months)	Number	Days	LOS	Number	Days	LOS	Number	Days	LOS
0–2	882	11,138	12,63	3550	30,643	8,63	146,181	1,113,071	7,61
3–5	758	5046	6,66	2530	13,926	5,5	21,200	111,387	5,25
6–11	1585	8920	5,63	4722	23,563	4,99	28,439	134,497	4,73
12–23	1874	8258	4,41	7035	28,077	3,99	44,225	182,522	4,13
24–35	632	2792	4,42	3282	12,217	3,72	30,613	114,294	3,73
36–47	224	966	4,31	1717	6408	3,73	26,864	89,420	3,33
48–59	121	458	3,79	1185	4174	3,52	24,733	72,549	2,93
Gender									
Boys	3427	21,313	6.22	13,338	66,539	4.99	182,322	1,013,967	5.56
Girls	2649	16,265	6.14	10,683	52,469	4.91	139,898	803,472	5.74
Total	6076	37,578	6,18	24,021	119,008	4,95	322,255	1,817,740	5,64

Number of hospitalizations (Number). Total days of stay (Days). Average length of stay in days (LOS)

84% of RVAGE hospitalizations occurred in children <2 years of age, being the group from 12 to 24 months the most affected. Boys were admitted more frequently for RVAGE (56.4%) and AGE (55.5%) than girls.

### Rotavirus vaccine impact

**Fig. 1:**
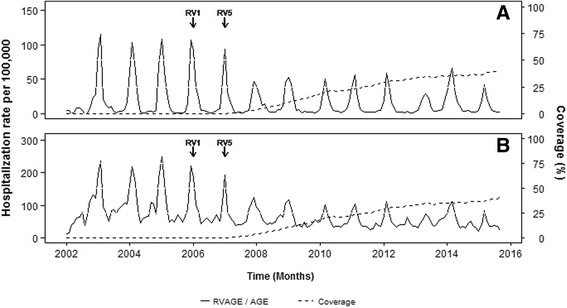
Monthly rate of **a** rotavirus AGE-associated hospitalizations, **b** AGE-associated hospitalizations and coverage in children < 5 years-old

The incidence of RVAGE-associated hospitalizations decreased markedly after the vaccines were marketed in 2007 (Fig. 1a). Similar reductions in all-cause AGE hospitalizations were observed (Fig. 1b). These declines were sustained over the years of the study. The vaccination coverage in our cohort reached 42.13%; however, high variability was shown among health departments. The suspension of both vaccines in 2010 was associated with a decline in the vaccination coverage (Additional file 1).

A general coverage-dependent response was observed, so the risk of RVAGE or AGE-hospitalizations decreased with increasing vaccine coverage (Table 2). With a vaccination coverage ≥40%, the rotavirus-coded hospitalization risk decreased to 67% (55-67), 71% (58-81) and 68% (18-92) in children 0, 1 and 4 years of age, respectively (Table 2). Even with vaccination coverage ˂20%, a reduction of 37% and 45% in children with 0 and 1 years of age, respectively, was found.

**Table 2:** Adjusted Relative Risk (RR) of RVAGE and AGE-associated hospitalizations depending on the vaccination coverage

	RVAGE					AGE				
Age (years)	0	1	2	3	4	0	1	2	3	4
RV vaccine coverage										
0%	1	1	1	1	1	1	1	1	1	1
1–19%	0,63 (0,49 – 0,79)	0,55 (0,4 – 0,74)	0,87 (0.56 – 1.31)	1.34 (0.63 – 2.50)	0.58 (0.21 – 1.26)	0.77 (0.69 – 0.86)	0.74 (0.64 – 0.85)	0.80 (0.68 – 0.94)	0.9 (0.72 – 1.12)	1 (0.76 – 1.27)
20–39%	0,47 (0,35 – 0,60)	0,3 (0,21– 0,42)	0,97 (0.55 – 1.54)	0.71 (0.30 – 1.36)	0.45 (0.16 – 1.07)	0.67 (0.59 – 0.75)	0.55 (0.47 – 0.64)	0.73 (0.59 – 0.88)	0.77 (0.59 – 1,00)	0.91 (0.65 – 1.22)
≥ 40%	0,33 (0,23 – 0,45)	0,29 (0.19 – 0.42)	0.63 (0.34 – 1.08)	0.47 (0.17 – 1.03)	0.32 (0.08 – 0.82)	0.68 (0.58 – 0.80)	0.61 (0.51 – 0.73)	0.64 (0.50 – 0.81)	0.67 (0.49 – 0.90)	0.82 (0.56 – 1.14)

(95% CrI)

*Bayesian mixed Poisson regression model was adjusted by gender, time, time in post-vaccination period, month of the year, health department and total hospitalization rate except AGE

Overall, similar results were found for all AGE-coded hospitalizations, consistent with an impact of vaccination (Table 2). In this case, the risk of hospitalization decreased in all age groups until the age of 4.

### Burden of hospitalizations attributable to AGE

The estimated number of days of hospitalization attributable to AGE and RVAGE in the period studied was 119,008 and 6076 days, respectively (Table 1). Children <2 years of age accounted for almost 90% and 80% of the total number of days of hospitalizations for rotavirus and all-cause AGE, respectively and RVAGE cases had about 50% longer average length of stay (LOS) in these groups than in the older groups (Table 1).

Overall, the number of days of hospitalization for AGE decreased between 11 and 30% after vaccines licensure (Table 3). These figures represented hospital costs savings of more than EUR 6 million per 10^5^ children in 7 years in The Region of Valencia.

**Table 3:** Cost reduction of AGE-hospitalizations in years after rotavirus vaccines licensure compared to the pre- vaccination period (2003-2006) studied among children <5 years old

		AGE-hospitalization		
Year	Rate^a^	Rate reduction (%)^b^	Costs (€)^a^	Costs reduction (€)^a^
2003-2006	6830,225	-	3,716,879,6	-
2008	4297,2	11	3,307,414,9	408,781,6
2009	3201,5	29	2,638,499,6	1,077,697,0
2010	3152,6	26	2,749,985,5	966,211,1
2011	3152,4	30	2,601,337,6	1,114,859,0
2012	3281,5	27	2,712,823,5	1,003,373,1
2013	2964,4	20	2,972,957,2	743,239,3
2014	3463,7	21	2,935,795,3	780,401,3

^a^Data presented per 10^5^ persons

Days of hospitalization per 10^5^ persons (Rate)

Average Rate and Costs data are presented in the pre-vaccination period (2003-2006)

^b^Rate reduction was estimated by the negative binomial model

2007 was considered as a transition year

## Discussion

Our findings showed that even a low rotavirus vaccination coverage had relevant impact in the reduction RVAGE-hospitalizations and in all-cause AGE hospitalizations in young children in Valencia. We were able to demonstrate, for the first time, a coverage-dependent response in all age groups, demonstrating that, in this ecological study, the impact is attributable specifically to the vaccine. The most dramatic reduction in rotavirus hospitalizations occurred in children <2 years old, which is the group most affected by rotavirus (84% of all the RVAGE hospitalization).

There was a decrease in hospitalizations in all age groups, although in the older groups it is less consistent, probably due to their low RVAGE hospitalization rate (between 10 and 40 per 100.000 inhabitants, 10 times lower that of the <1 year of age).

Other ecological studies have shown a decrease in hospitalizations for RVAGE or AGE after vaccination licensure [4–6, 12]; however, these do not control for factors that may modify hospitalizations practices. As the vaccines licensure occurred around 2007, when the economic crisis started in Spain, changes in admission policies could be expected to save resources and therefore not all the hospitalizations changes could be attributable to the vaccine. To offset this effect, we controlled the model for the total hospitalizations in this age group. A second advantage of our model is that we were able to show a clear dose-response between vaccination coverage and hospitalizations. Therefore, although we are aware that perhaps only admission policies for diarrhoea and not for the rest could have changed, the overall potential bias of this ecological study is minimized.

On the other hand, different studies around the world showed a high variability in the reductions in RVAGE-hospitalizations [4, 12, 20, 21], ranging from 14% to 94%. In EEUU, for instance, these reductions were between 60% and 94% in children <5-years old with a constant vaccination coverage of 63% [9]. Differences in study designs, year of the study, data sources, geographic areas and vaccine coverage, are limitations for comparisons among studies. Different studies in Spain with equivalent vaccination coverage, showed RVAGE-hospitalizations reductions ranging from 10% to 57% in children <1 year of age [4, 22] and 14% to 44.5%, in children less than 5 years of age [4, 6, 12, 22]. The strength of the model used in this study and the novelty to analyse the impact by vaccination coverage over a 14-year period may have allowed us to obtain more accurate results.

When analysing rotavirus epidemiology, the diagnostic test used and guidelines to test for etiological diagnosis should be taken into consideration [1]. During the study period, especially after vaccination licensure, all hospitals in the Region used immunochromatography of different trademarks [23] and these had different sensitivities and specificities. Moreover, the proportion of cases tested varied among hospitals and also with time [24], hindering the assessment of the epidemiology of RVAGE. In the same line, as the diagnosis of rotavirus does not modify the management of the AGE, the specific diagnostic assay is not always recommended. Therefore, as other studies pointed out [4], there is a potential under-estimation of the real rotavirus hospital burden in Spain and we consider that impact analysis of the RV vaccine should always consider the impact on all-cause AGE.

Nevertheless, some limitations of our study should be highlighted. Rotavirus vaccines are not included in the official immunization schedule and this may suggest differences between rotavirus vaccinees and non-vaccinees with respect to socio-economic conditions and health seeking behaviour [4]. In addition, we cannot discard the possibility that some doses could have been missed or administered in private vaccination centres not using SIV. Nonetheless, a recent study showed that, most of the rotavirus vaccine doses (83%) distributed in Valencia were registered in SIV as administered in children aged <1 year [25].

On the other hand, RV1 and RV5 were used concurrently until 2010, therefore from 2010 to June 2016, the only rotavirus vaccine available in Spain was RV5, and our results will have limited value to estimate the impact of RV1. Thus, the study assumed that most of the impact was due to RV5.

Finally, it is important to highlight that this is the first national study published that estimates the reduction (more than EUR 6 million per 10^5^ children in 7 years) in health care costs from all-AGE hospitalizations following vaccine implementation. However, other factors would be required for a full analysis of the cost-effectiveness of vaccination, particularly; the cost of the vaccine program and indirect cost savings from prevention of lost productivity. It has been estimated that when a child is sick by rotavirus, a parent has to miss work an average of 4 days, due to child care at home or in the hospital [16]. In addition, other expenses for the treatment of symptoms such us serum or diapers were not included.

## Conclusions

In summary, our findings demonstrate that rotavirus vaccines had a specific coverage-dependent response impact in the risk of hospitalization for rotavirus in children <5 years. Overall, an important decline in both RVAGE and AGE-hospitalizations in all the groups studied, especially in children less than 2 years old. This reduction represents a savings for the national health system of more than EUR 6 million per 10^5^ children in 7 years in The Region of Valencia. The model used reassures that the estimated impact is due to the vaccination and not to other external factors.

## Additional file

Additional file 1: Vaccine coverage in The Region of Valencia (Spain) by age group during the study period (2002-2015). Vaccine coverage was defined as the proportion of the study population vaccinated with at least one dose of RV1 or RV5, with no distinction between vaccines. However, in 2010 no new batches of vaccine were released into the market for 5 months due to the detection of circovirus in both vaccines within that period. (DOC 96 kb)

## Abbreviations

AGE: All-cause acute gastroenteritis
CMBD: Spanish hospital discharge database
CrI: Credible intervals
NHS: National Health System
RR: Risk ratios
RV1: Rotarix® (GlaxoSmithKline Biologicals, Rixensart, Belgium)
RV5: RotaTeq® (Merck & Co., Inc., West Point, PA, USA)
RVAGE: Rotavirus acute gastroenteritis
SIP: Valencia’s administrative population-based database
SIV: Valencia’s Vaccine Information System

## References

[1] Patel M WM, Cortese M, Gentsch J, Glass R and Parashar U. Generic protocol for monitoring impact of rotavirus vaccination on gastroenteritis disease burden and viral strains. Immunization, Vaccines and Biologicals [Internet]. 2008; (Immunization, Vaccines and Biologicals). Available from: http://apps.who.int/iris/bitstream/10665/69913/1/WHO_IVB_08.16_eng.pdf.

[2] The paediatric burden of rotavirus disease in Europe. Pediatric ROTavirus European CommitTee (PROTECT). Epidemiol Infect. 2006;134(5):908–16. Epub 2006 Apr 4. 10.1017/S0950268806006091 PMC2870494 16650331

[3] Cortese MM, Parashar UD (2009). Prevention of rotavirus gastroenteritis among infants and children: recommendations of the advisory Committee on immunization practices (ACIP). MMWR Recomm Rep.

[4] Gil-Prieto R, Gonzalez-Escalada A, Alvaro-Meca A, Garcia-Garcia L, San-Martin M, Gonzalez-Lopez A (2013). Impact of non-routine rotavirus vaccination on hospitalizations for diarrhoea and rotavirus infections in Spain. Vaccine.

[5] Martinon-Torres F, Martinon-Torres N, Bouzon Alejandro M, Redondo Collazo L, Pertega-Diaz S, Seoane-Pillado MT (2012). Acute gastroenteritis hospitalizations among children aged < 5 years before and after introduction of rotavirus vaccines: a hospital-based surveillance study in Galicia. Spain. Hum Vaccin Immunother..

[6] Redondo O, Cano R, Simon L (2015). Decline in rotavirus hospitalizations following the first three years of vaccination in castile-la Mancha. Spain Hum Vaccin Immunother.

[7] Van Damme P, Giaquinto C, Huet F, Gothefors L, Maxwell M, Van der Wielen M (2007). Multicenter prospective study of the burden of rotavirus acute gastroenteritis in Europe, 2004-2005: the REVEAL study. J Infect Dis.

[8] Perez-Vilar S, Diez-Domingo J, Lopez-Lacort M, Martinez-Ubeda S, Martinez-Beneito MA (2015). Effectiveness of rotavirus vaccines, licensed but not funded, against rotavirus hospitalizations in the Valencia region. Spain BMC Infect Dis.

[9] Leshem E, Moritz RE, Curns AT, Zhou F, Tate JE, Lopman BA (2014). Rotavirus vaccines and health care utilization for diarrhea in the United States (2007-2011). Pediatrics.

[10] Forster J, Guarino A, Parez N, Moraga F, Roman E, Mory O (2009). Hospital-based surveillance to estimate the burden of rotavirus gastroenteritis among European children younger than 5 years of age. Pediatrics.

[11] Davey HM, Muscatello DJ, Wood JG, Snelling TL, Ferson MJ, Macartney KK (2015). Impact of high coverage of monovalent human rotavirus vaccine on emergency department presentations for rotavirus gastroenteritis. Vaccine.

[12] Gimenez Sanchez F, Nogueira EJ, Sanchez Forte M, Ibanez Alcalde M, Cobo E, Angulo R (2016). Impact of vaccination uptake on hospitalizations due to rotavirus acute gastroenteritis in 2 different socioeconomic areas of Spain. Hum Vaccin Immunother..

[13] Economou K, Kentikelenis, Sisouras and Maresso. The impact of the finaltial crisis on the health system and health in Greece. World Health organization; 2014. http://www.euro.who.int/__data/assets/pdf_file/0007/266380/The-impact-of-the-financial-crisis-on-the-health-system-and-health-in-Greece.pdf.

[14] Diez Domingo J, Patrzalek M, Cantarutti L, Arnould B, Meunier J, Soriano-Gabarro M (2012). The impact of childhood acute rotavirus gastroenteritis on the parents' quality of life: prospective observational study in European primary care medical practices. BMC Pediatr.

[15] Parashar UD, Gibson CJ, Bresee JS, Glass RI (2006). Rotavirus and severe childhood diarrhea. Emerg Infect Dis.

[16] Alvarez Aldean J, Aristegui J, Lopez-Belmonte JL, Pedros M, Sicilia JG (2014). Economic and psychosocial impact of rotavirus infection in Spain: a literature review. Vaccine.

[17] Ministerio de Sanidad PSeI. Informe Anual del Sistema Nacional de Salud2011. Available from: http://www.msssi.gob.es/organizacion/sns/planCalidadSNS/pdf/equidad/informeAnualSNS2011/05_INFORME_SNS_2011_ESPANYOL.pdf.

[18] Morant-Talamante N, Diez-Domingo J, Martinez-Ubeda S, Puig-Barbera J, Aleman-Sanchez S, Perez-Breva L (2013). Herpes zoster surveillance using electronic databases in the Valencian community (Spain). BMC Infect Dis.

[19] Fiscal Measures, Administrative and Financial Management and Organization of the Generalitat valenciana, DOGV 2014/11805 (2014). http://www.dogv.gva.es/datos/2014/12/29/pdf/2014_11805.pdf.

[20] Gagneur A, Nowak E, Lemaitre T, Segura JF, Delaperriere N, Abalea L (2011). Impact of rotavirus vaccination on hospitalizations for rotavirus diarrhea: the IVANHOE study. Vaccine.

[21] Karafillakis E, Hassounah S, Atchison C (2015). Effectiveness and impact of rotavirus vaccines in Europe, 2006-2014. Vaccine.

[22] Martinon-Torres F, Aramburo A, Martinon-Torres N, Cebey M, Seoane-Pillado MT, Redondo-Collazo L (2013). A reverse evidence of rotavirus vaccines impact. Hum Vaccin Immunother.

[23] Lopez-Lacort M, Collado S, Diez-Gandia A, Diez-Domingo J (2016). Rotavirus, vaccine failure or diagnostic error?. Vaccine.

[24] V L-M. ¿Qué aporta el diagnóstico etiológico de las enfermedades comunes infantiles? : Catholic University of Valencia “San Vicente Mártir”; 2016.

[25] Perez-Vilar S, Diez-Domingo J, Gomar-Fayos J, Pastor-Villalba E, Sastre-Canton M, Puig-Barbera J (2014). Post-licensure passive safety surveillance of rotavirus vaccines: reporting sensitivity for intussusception. An Pediatr (Barc).

