# Supplementary figures and images for: Long-term impact of self-financed rotavirus vaccines on rotavirus-associated hospitalizations and costs in the Valencia Region, Spain
Source: BMC Infect Dis. 2017 Apr 11;17:267. doi: 10.1186/s12879-017-2380-2 (PMC5387249; doi:10.1186/s12879-017-2380-2)

Additional file 1. Evolution of the vaccination coverage by age group during the study period (2002-2015).


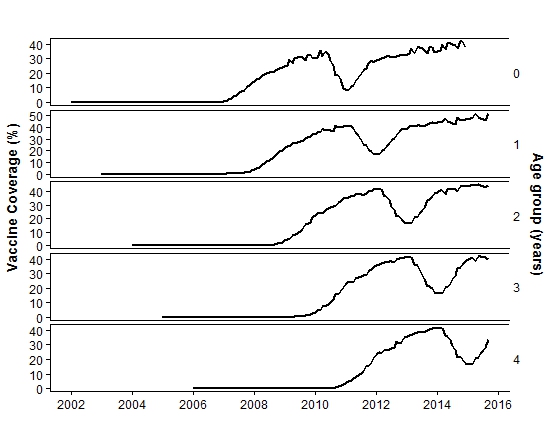

Supplement: Additional file 1: — Vaccine coverage in The Region of Valencia (Spain) by age group during the study period (2002-2015). Vaccine coverage was defined as the proportion of the study population vaccinated with at least one dose of RV1 or RV5, with no distinction between vaccines. However, in 2010 no new batches of vaccine were released into the market for 5 months due to the detection of circovirus in both vaccines within that period. (DOC 96 kb) [file 12879_2017_2380_MOESM1_ESM.doc]
